# Region‐specific effects of oestradiol on adipose‐derived stem cell differentiation in post‐menopausal women

**DOI:** 10.1111/jcmm.13011

**Published:** 2016-11-15

**Authors:** Kimberly A. Cox‐York, Christopher B. Erickson, Rocio I Pereira, Daniel H. Bessesen, Rachael E. Van Pelt

**Affiliations:** ^1^ Department of NutritionColorado State UniversityFort CollinsCOUSA; ^2^ Department of MedicineDivision of Geriatric MedicineUniversity of Colorado Anschutz Medical CampusAuroraCOUSA; ^3^ Department of MedicineDivision of Endocrinology, Metabolism and DiabetesUniversity of Colorado Anschutz Medical CampusAuroraCOUSA; ^4^ Denver Health and Hospital AuthorityDenverCOUSA

**Keywords:** adipose tissue, oestrogen, stem cells, menopause, adipogenesis

## Abstract

The goal of this study was to determine the effect of acute transdermal 17β‐oestradiol (E_2_) on the adipogenic potential of subcutaneous adipose‐derived stem cells (ASC) in post‐menopausal women. Post‐menopausal women (*n* = 11; mean age 57 ± 4.5 years) were treated for 2 weeks, in a randomized, cross‐over design, with transdermal E_2_ (0.15 mg) or placebo patches. Biopsies of abdominal (AB) and femoral (FEM) subcutaneous adipose tissue (SAT) were obtained after each treatment and mature adipocytes were analysed for cell size and ASC for their capacity for proliferation (growth rate), differentiation (triglyceride accumulation) and susceptibility to tumour necrosis factor alpha‐induced apoptosis. Gene expression of oestrogen receptors α and β (ESR1 and ESR2), perilipin 1 and hormone‐sensitive lipase (HSL), was also assessed. In FEM SAT, but not AB SAT, 2 weeks of E_2_ significantly (*P* = 0.03) increased ASC differentiation and whole SAT HSL mRNA expression (*P* = 0.03) compared to placebo. These changes were not associated with mRNA expression of oestrogen receptors α and β, but HSL expression was significantly increased in FEM SAT with transdermal E_2_ treatment. Adipose‐derived stem cells proliferation and apoptosis did not change in either SAT depot after E_2_ compared with placebo. Short‐term E_2_ appeared to increase the adipogenic potential of FEM, but not AB, SAT in post‐menopausal women with possible implications for metabolic disease. Future studies are needed to determine longer term impact of E_2_ on regional SAT accumulation in the context of positive energy imbalance.

## Introduction

Loss of endogenous ovarian hormones is thought to play a role in menopause‐related increases in subcutaneous adipose tissue (SAT) accumulation, particularly shifts in SAT distribution from the femoral (FEM) to abdominal (AB) region. This gynoid‐to‐android redistribution is proposed to contribute to the rise in cardiometabolic disease risk observed in women at the time of menopause 1, 2. The decline in endogenous sex hormones (in particular 17β‐oestradiol, E_2_) likely impacts SAT accumulation indirectly through central mechanisms that regulate energy intake and expenditure 3, but there may also be a direct effect on SAT expandability at the cellular level.

Subcutaneous adipose tissue hypertrophy is determined by the capacity for committed adipocytes to store triglycerides, while the capacity for SAT to expand *via* hyperplasia is dependent on the proliferation and differentiation (adipogenic potential) of adipocyte precursor cells (adipose‐derived stem cells; ASC) 4, 5. Adipose‐derived stem cells isolated from discrete human adipose tissue regions (visceral, omental, subcutaneous) and maintained in culture appear to vary in their capacity for adipogenesis, 6, 7, 8 where proliferation is reportedly greater in SAT than visceral adipose tissue (VAT) and differentiation is either greater or not different in SAT relative to VAT.

Animal and cell models support E_2_‐mediated regulation of adipogenic potential. *In vitro* E_2_ treatment decreased lipid accumulation in adipocyte precursor cells derived from rat SAT 9, 10 and mouse bone marrow cells 11, as well as in 3T3 mouse fibroblasts 10. Human ASC treated *in vitro* with E_2_ also exhibited altered adipogenic potential 12, 13, and 3 months of E_2_ treatment altered the expression of genes central to lipogenesis (*e.g*. SCD1, FAS, ACC1) in SAT from post‐menopausal women 14. Administration of *in vivo* compounds has previously been shown to modulate *ex vivo* adipogenic potential of ASC, suggesting that ASC maintain the attributes of their origin 15. However, to our knowledge, the adipogenic potential of ASC isolated from human participants treated with E_2_ has never been studied.

We previously reported that in post‐menopausal women, acute E_2_ treatment resulted in decreased cell size and increased cell number (hyperplasia) in FEM, but not AB SAT 16. We postulated that this difference may be driven by the adipogenic potential of the ASC. We employed a randomized, cross‐over design to treat post‐menopausal women with transdermal E_2_ or placebo to study the capacity for proliferation, differentiation and susceptibility to apoptosis in primary ASC isolated from FEM and AB SAT.

## Materials and methods

### Volunteers

Study volunteers were: (*i*) aged 45–60 years; (*ii*) post‐menopausal (no menses ≥12 months or history of bilateral ovariectomy and FSH >30 IU/l); (*iii*) with body mass index >20 and <30 kg/m^2^; (*iv*) weight stable (±2 kg in past 2 months); (*v*) non‐smokers; (*vi*) sedentary to moderately active; and (*vii*) not using any type of hormone therapy or oral contraceptives. Exclusion criteria included: diabetes, hypertriglyceridaemia (TG >400 mg/dl), medications known to affect lipid metabolism, elevated resting blood pressure (>140 mmHg systolic, >90 mmHg diastolic) and any conditions in which E_2_ treatment would be contraindicated (*e.g*. increased risk of blood clots, stroke, heart disease and breast cancer). All participants provided written informed consent to participate in the study, in accordance with the approved guidelines of the Colorado Multiple Institutional Review Board.

### Body composition assessment

Total and regional (trunk, leg) fat mass and fat‐free mass were assessed at baseline by dual‐energy x‐ray absorptiometry (DXA, Hologic Discovery W, software version 11.2; Hologic, Inc., Marlborough, MA) as previously described 17.

### Energy balance

Prior to enrollment and throughout the testing, participants were required to maintain their current body weight ±2 kg and to refrain from exercise for the 3 days leading up to the biopsy visit.

### E_2_ administration

Each participant acted as their own control. Participants were randomly assigned to wear either identical placebo (no hormone) or E_2_ (0.15 mg; 3 × 0.005 mg ea.) transdermal patches for 2 weeks. Patches were placed on the flank, just above the buttocks. Following a 6–8 week‐wash‐out period, participants were switched to the alternate treatment. Subcutaneous adipose tissue biopsies were obtained at the end of each treatment from the FEM (thigh) and AB (lateral umbilicus) regions as described below.

### Blood work

Fasting serum samples were stored at −80°C and analysed in batch. Glucose was determined enzymatically (Beckman Coulter; Brea, CA); insulin, leptin by radioimmunoassay (EMD Millipore; Darmstadt, Germany); and oestradiol and sex hormone‐binding globulin by chemiluminescence (Beckman Coulter; Brea, CA). Sensitivity and precision details can be found at http://cctsi.ucdenver.edu/Research-Resources/CTRCs/Pages/Assays.aspx.

### Adipose tissue biopsies

Aspiration biopsies of subcutaneous AB and FEM SAT were collected in the fasting state at both visits, and performed with a mini‐liposuction technique as previously described 18. Approximately 0.5–1.5 g of tissue was obtained from each biopsy. This was sufficient material to complete cell sizing, mRNA expression and cellular dynamics studies for most participants (*n* = 11) as indicated in figure legends.

### Mature adipocyte size

Immediately after collection, 50 mg of SAT was collagenase digested. Mature adipocytes were stained and cell size determined with Cell Counting Analysis Program (Mayo Clinic, Rochester, MN, USA) as previously described 19, 20.

### Adipose‐derived stem cell isolation and analysis

Adipose‐derived stem cells were isolated from AB and FEM biopsy samples as described previously 21. Briefly, fresh SAT (0.5–1.5 g) was collagenase digested, filtered, then centrifuged to pellet the stromal vascular (SV) fraction. The SV was treated with erythrocyte lysis buffer, then seeded in growth medium (α‐MEM with 10% foetal bovine serum (FBS), penicillin: 100 U/ml, streptomycin: 100 μg/ml). Media was changed after 24 hrs. Once the cells reached ~80% confluence, they were detached, passaged twice and frozen in an isopropanol container before storing in liquid nitrogen. Cells were revived from frozen and analysed in batch—both depots and both study days were analysed together for each participant. This method has been shown to yield 70% adipogenic precursor cells (CD34^+^ CD31^−^), and only a small percentage of monocytes/macrophages (CD11b/c+) and endothelial cells (CD34^+^ CD31^+^) after an initial culture of 3 hrs 22. The overnight culture yields an even more pure population 23.

### Differentiation

Frozen cells (passage 3–4) were revived from frozen in fresh growth medium and plated in triplicate 12‐well plates (5000 c/cm^2^). At confluence, the medium was switched to either low glucose maintenance medium (controls; DMEM‐low glucose, 10% FBS, 2 mM L‐glutamine, 5 μg/ml gentamycin) or commercial differentiation‐inducing medium (differentiated; Stem‐Pro Adipogenesis Differentiation kit, cat. A10070‐01; Life Technologies; Carlesbad, CA). After 12–14 days, control and differentiated cells were stained for lipid with Oil Red O for 15 min, followed by multiple rinses with ddH_2_O as previously described 24. Oil Red O was then extracted with isopropanol and absorbance measured at 569 nm. Differentiation was also quantified using AdipoRed Assay Reagent (Lonza, Walkersville, MD) per manufacturer's instructions and fluorescence was detected at (485_EX_/572_EM_). Differentiation values are reported as a percent of undifferentiated, control cells.

### Proliferation

Proliferation was assessed *via* bioreduction of a novel tetrazolium compound by metabolically active cells at several time‐points across the growth phase using CellTiter 96 reagent (cat. G3580; Promega Corp.; Madison, WI). Briefly, cells were seeded in triplicate in a 96‐well plate (1000 cells/well; 3000 c/cm^2^). After overnight incubation the proliferation reagent was added, the plate was incubated at 37°C for 1 hr, and absorbance was read at 490 nm. Proliferation was monitored every 24 hrs in continuously cultured cells up to 72 hrs after plating. Values are presented as daily growth rate (absorbance). Proliferation plateaued between 48 and 72 hrs, therefore the slope from 0 to 48 hrs was calculated for each sample.

### Apoptosis

Apoptosis was quantified relative to total viability using the ApoLivGlo kit (cat. G6410; Promega Corp.), a duplexed assay designed to measure viability (live‐cell protease activity) and apoptosis (caspase‐3/7 activation) in the same well. Cells were plated at 10^4^ cells/cm^2^ in opaque, 96‐well plates. After an overnight incubation in growth medium, cells were treated with tumour necrosis factor (TNF)‐α (10 nmol/l) and cyclohexamide (10 μg/ml) to induce apoptosis. After 6 hrs of treatment, the viability reagent was added, incubated for 30 min at 37°C, and fluorescence read at 400_EX_/505_EM_. Subsequently, apoptosis reagent was added to the same wells, incubated at room temperature for 30 min and luminescence was measured.

### mRNA quantification (qPCR)

Total RNA was isolated from 50 to 100 mg of whole adipose tissue using the RNeasy Lipid Mini Kit (Qiagen Inc.; Hilden, Germany). One microgram of RNA was (*i*) analysed and quantitated using Experion (Bio‐Rad; Hercules, CA), (*ii*) reverse transcribed using iScript (Bio‐Rad), and (*iii*) analysed by qPCR in duplicate using iQ SYBR Supermix (Bio‐Rad) on an iQ5 Real‐Time PCR Detection System (Bio‐Rad) along with a no‐template control per gene. Reference gene ribosomal protein L13a (RPL13A) was used for normalization. The efficiencies of target and reference genes were approximately equal, and RPL13A expression was not different between groups (overall standard deviation 0.49). Primers (Bio‐Rad) sequences can be found in Table 1. The relative change in mRNA expression in response to E_2_ treatment was calculated as 2‐^ddCt^.

**Table 1:** Subject characteristics (*n* = 11)

Variable	Placebo	Oestradiol
Age (years)	57 ± 4	
Years past menopause	7 ± 6	
Weight (kg)	64.4 ± 9.1	
BMI (kg/m^2^)	25.1 ± 2.8	
Fat‐free mass (kg)	41.0 ± 5.4	
Fat mass (kg)	23.7 ± 5.7	
Trunk fat mass (kg)	11.3 ± 4.1	
Leg fat mass (kg)	9.0 ± 1.7	
Fasting glucose (mg/dl)	87.0 ± 1.9	84.4 ± 2.2
Fasting insulin (μU/ml)	10.7 ± 1.1	9.6 ± 1.0
Oestradiol (pg/ml)	14.3 ± 2.3	115.6 ± 17.2**
Sex hormone‐binding globulin (nm/l)	50.8 ± 5.1	53.6 ± 5.1
Leptin (ng/ml)	14.9 ± 3.3	17.4 ± 3.8*
Adiponectin (μg/ml)	17.7 ± 4.3	17.7 ± 3.1

Mean ± SD; *P = 0.02, **P < 0.001 compared to placebo.

### Statistical analyses

All data were examined using descriptive statistics (mean, median, S.D., range) and graphical summaries (boxplots, scatterplots). Paired *t‐*tests were used to evaluate changes in response to E_2_ compared with placebo. Depot‐specific differences were analysed using unpaired *t*‐tests. Analyses were done using IBM SPSS Statistics (v22; IBM, Inc.; Armonk, NY).

## Results

### Participant characteristics

Study participants were healthy, non‐obese (BMI 20–29 kg/m^2^) post‐menopausal women (aged 51–59 years) ranging from 1 to 15 years since their final menses (Table 1). Compared to placebo, 2 weeks of transdermal E_2_ treatment significantly increased plasma E_2_ (14.3 ± 2 *versus* 115.6 ± 17 pg/ml; *P* < 0.001). Plasma leptin was also significantly increased with E_2_ relative to placebo (*P* = 0.02).

### Adipose tissue cellularity

**Figure 1:**
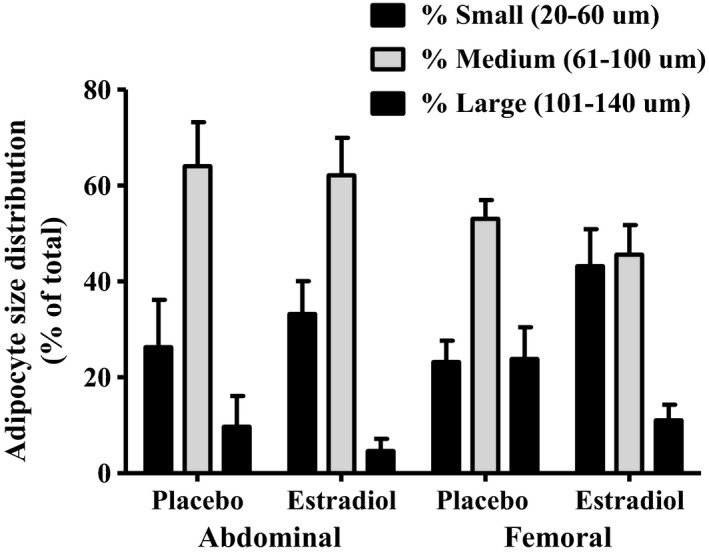
Change in adipocyte size. Change (mean ± S.E.M.) in proportion of small (20–60 μm; *P* = 0.09), medium (61–100 μm, *P* = 0.07) and large (101–140 μm; *P* = 0.07) in femoral (FEM) and abdominal AB SAT following 2 weeks of transdermal oestradiol (E_2_) compared with placebo treatment; *n* = 8 AB, 11 FEM.

There were no differences in cell diameter or cell size distribution between AB and FEM SAT under placebo‐treated conditions (Fig. 1). Short‐term E_2_ treatment did not significantly change mean adipocyte size in the FEM SAT depot (61 ± 19 E_2_ *versus* 76 ± 14 μm placebo; *P* = 0.09), however, there was a trend towards an increase in the proportion of small (20–60 μm; *P* = 0.07) and decreased proportion of large (100–140 μm; *P* = 0.07) adipocytes. Adipocyte size in the AB SAT depot did not change in response to E_2_ (*P* = 0.51).

**Table 2:** mRNA analysis; qPCR primer sequences

GenBank no.	Gene	5′ primer	3′ primer
NM_002666	PLIN1	GGAAGAATTGGAGACTGAGGAG	GGTCTTCTGCAGGGTATGTG
NM_005357	HSL	AACCAGTGCTCGGAATCACAGACA	AGTCACCAGCGACTGTGTCATTGT
NM_000125	ERα	AGATCTTCGACATGCTGCTGGCTA	AGACTTCAGGGTGCTGGACAGAAA
NM_001437	ERβ	TTGGTTTGGGTGATTGCCAAGAGC	ATGTTGAGCAGATGTTCCATGCCC
NM_012423	RPL13A	CCTGGAGGAGAAGAGGAAAGAGA	TTGAGGACCTCTGTGTATTTGTCAA

### Adipogenic potential

**Figure 2:**
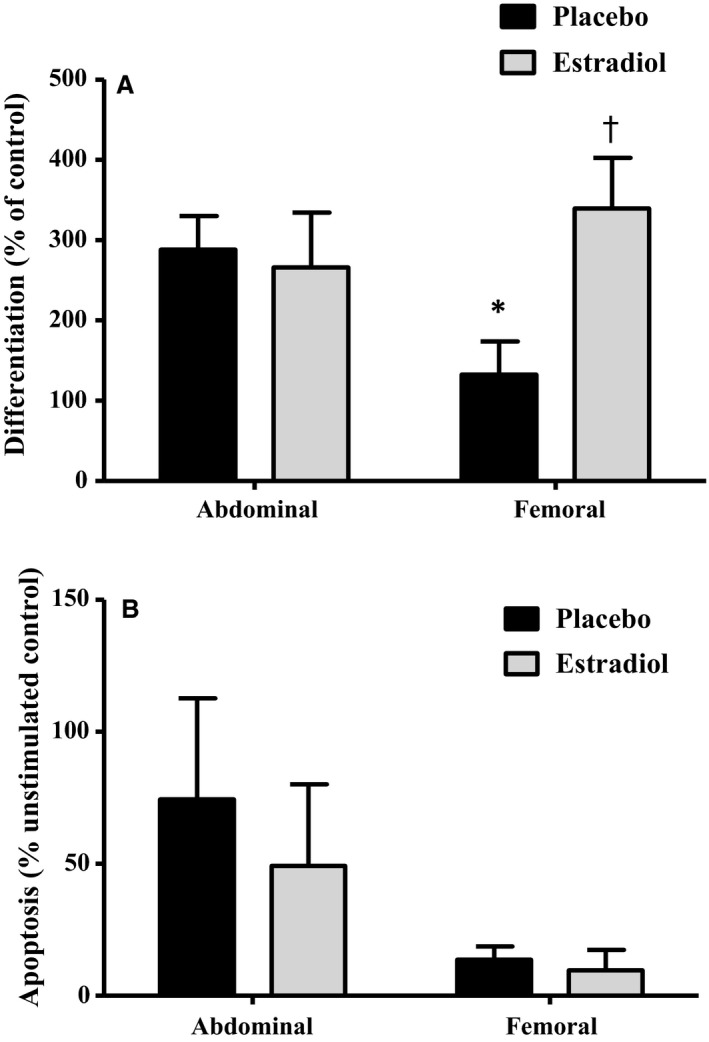
ASC differentiation and apoptosis. (**A**) Adipose‐derived stem cell (ASC) differentiation [triglyceride accumulation; *n* = 6–8 abdominal (AB), 8–10 femoral (FEM)]. *P = 0.02 FEM *versus* AB SAT in placebo condition; ^†^ *P* = 0.03 FEM SAT placebo *versus* E_2_. (**B**) ASC susceptibility to TNFα‐induced apoptosis (*n* = 5/group) in FEM and AB subcutaneous adipose tissue following 2 weeks of transdermal oestradiol (E_2_) compared to placebo.

**Figure 3:**
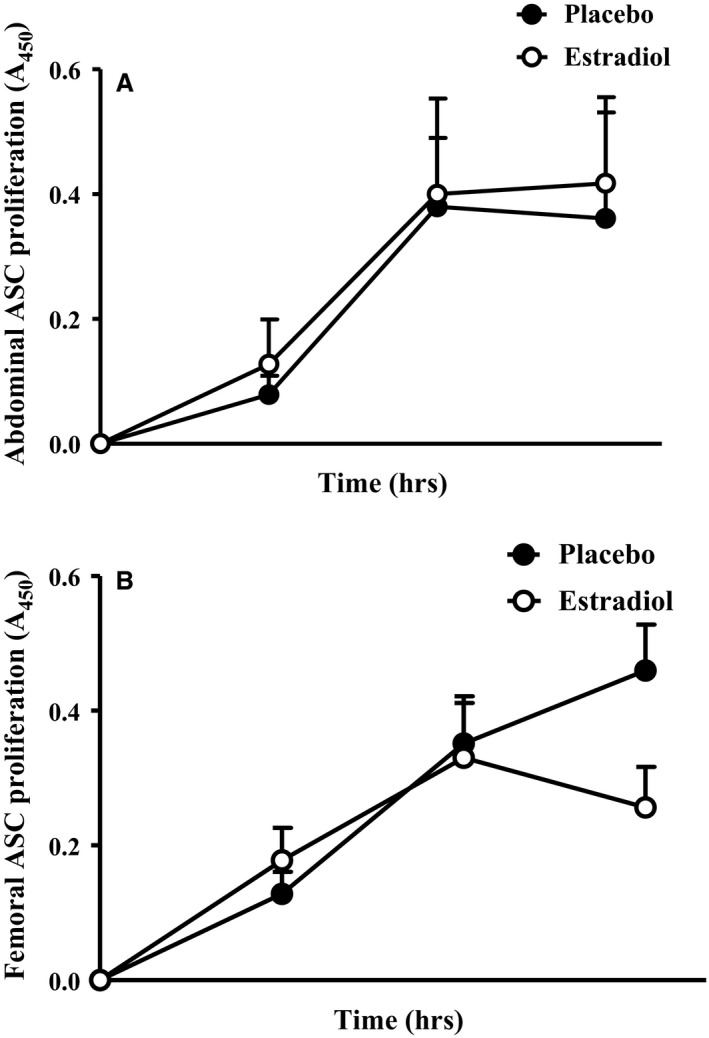
ASC proliferation. (**A**) Abdominal (AB) and (**B**) femoral (FEM) subcutaneous adipose‐derived stem cell (ASC) proliferation (A_450_) following 2 weeks of transdermal oestradiol (E_2_) or placebo; *n* = 10 AB,* n* = 9 FEM.

In the placebo condition, ASC from FEM SAT underwent significantly less differentiation than ASC from AB SAT (Fig. 2A; *P* = 0.02). Compared to placebo, E_2_ treatment significantly increased differentiation in ASC isolated from FEM SAT (*P* = 0.03), but not those isolated from AB SAT (*P* = 0.98). Proliferation was not different between SAT depots, or between E_2_ and placebo conditions (Fig. 3). While FEM SAT, compared to AB SAT, tended (*P* = 0.06) to be less susceptible to TNF‐α‐induced apoptosis under control conditions, there was no effect of E_2_ treatment in either region (Fig. 2B).

### Gene expression

**Figure 4:**
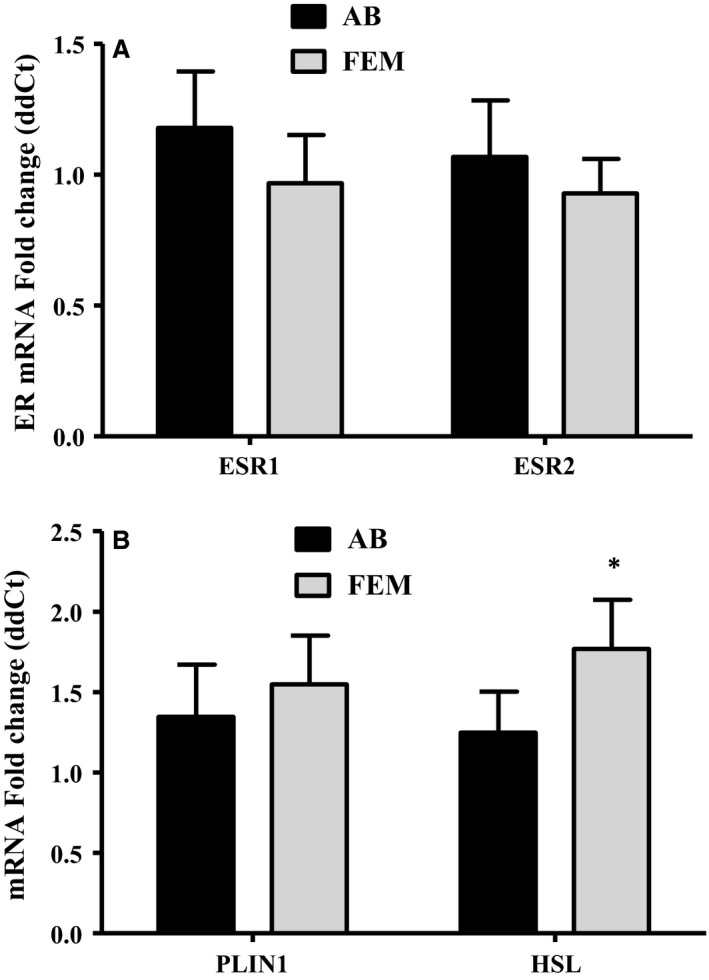
mRNA expression. (**A**) ESR1 (ERα) and ESR2 (ERβ). (**B**) Perilipin 1 (PLN1) and hormone‐sensitive lipase (HSL) mRNA relative expression (2‐ddCt) in abdominal (AB) and femoral (FEM) SAT with 2 weeks of transdermal E_2_ relative to placebo. *P<0.05

Transdermal E_2_ treatment did not significantly change mRNA expression of ERS1 and ERS2 in SAT from either FEM or AB regions (Fig. 4A). Expression of perilipin (PLIN1) mRNA was also unchanged, however, relative hormone‐sensitive lipase (HSL) expression (dCt) was significantly greater (*P* = 0.03) in FEM SAT after transdermal E_2_ treatment than after placebo treatment (Fig. 4B).

## Discussion

This study demonstrated that, compared with placebo, 2 weeks of transdermal E_2_ in post‐menopausal women increased FEM, but not AB, ASC differentiation. This change was accompanied by a modest decrease in FEM adipocyte size and increased HSL mRNA expression, while rates of ASC proliferation and apoptosis were not altered by treatment with E_2_ compared with placebo. These preliminary observations suggest that E_2_ may increase the adipogenic potential of SAT in a regionally specific manner to influence SAT expansion.

Lower body SAT is associated with decreased risk for cardiometabolic disease 25 and smaller adipocytes are generally recognized as being more insulin sensitive and capable of expansion 26, 27, 28, 29. We recently reported that pre‐menopausal women have smaller adipocytes in FEM SAT than post‐menopausal women, and that 2 weeks of E_2_ treatment in post‐menopausal women resulted in a reduction in mean adipocyte size in FEM SAT 16. In the current study, transdermal E_2_ treatment also resulted in a consistent trend towards smaller adipocytes in FEM SAT. This trend did not reach significance, perhaps because of the smaller participant number (*n* = 11) and relatively wide range in time since menopause. We did not observe a change in AB SAT cell size, indicating depot‐specific E_2_ regulation of adipose tissue cellularity. E_2_ treatment may mediate SAT cell size, and thereby SAT expansion by modifying aspects of lipolysis 3, 30 and/or adipogenesis.

Adipose tissue expansion is largely dependent on the adipocyte progenitor population to proliferate (multiply) and differentiate (take up and store lipid) into mature adipocytes (adipogenesis). In our *ex vivo* model of ASC adipogenesis, we did not observe a difference in ASC proliferative capacity in either AB or FEM SAT collected from post‐menopausal women treated with E_2_ and placebo. These results are in contrast to the increased proliferation of AB ASC observed when cells from pre‐ and post‐menopausal women are treated with E_2_ *in vitro* 12, 13. On the other hand, removal of endogenous estrogen through ovariectomy in ASC‐specific ER? knock‐out mice was shown to increase adipocyte progenitor proliferation 31. Together these results highlight potential differences in ASC proliferation when E_2_ is given *in vivo versus in vitro* and based on prevailing tissue ER expression.

Compared to age‐matched men, pre‐menopausal women have a greater number of immature (early differentiated; aP2^+^/CD68^−^) adipocytes, particularly in the FEM compared to AB depot, supporting a role for sex hormones in regional adipocyte recruitment 23. We found that 2 weeks of transdermal E_2_, compared with placebo, increased FEM, but not AB, ASC differentiation in post‐menopausal women. We are unaware of other human *ex vivo* studies with which to compare our results. However, one study of *in vitro* E_2_ treatment demonstrated that AB ASC isolated from a single female participant resulted in a dose‐dependent increase in ASC differentiation 32. Cell line and animal studies are mixed in this regard, with some reporting increased 9, 32 and others decreased 11, 33, 34, 35 differentiation with E_2_ treatment. As with proliferation rates it is likely that the effect of E_2_ on differentiation depends on whether it is administered *in vivo* or to cells *in vitro* and the ER expression profile. We observed lower differentiation in FEM, compared to AB ASC in the placebo treated condition. This regional difference has been observed previously 23, 36, 37 and is retained in cells passaged for multiple generations 6. Recent evidence suggests that precursors from different regions express variable levels of developmental genes (*i.e*. HOX) and have different developmental origins (reviewed in 38).

In addition to proliferation and differentiation, adipose tissue mass also depends on progenitor cell death. Regional differences in susceptibility of adipocyte precursors to apoptosis have been observed 21, but unlike differentiation, there do not appear to be sex differences 23. The effect of E_2_ on apoptosis has not been well studied, but Luo *et al*. recently showed that *in vitro* treatment of FEM ASC from pre‐menopausal women decreased serum starvation‐induced apoptosis 39. In contrast, we found no differences in the susceptibility of ASC to TNF‐α‐induced apoptosis in response to acute E_2_ treatment *in vivo*. This discrepancy is likely due to differences in the experimental design, i.e., *in vitro* application of E_2_ *versus ex vivo* susceptibility to apoptosis with *in vivo* E_2_ administration. It is also possible that age and menopausal status contributes to differential susceptibility of ASC to apoptosis as the participants in the *in vitro* study were young women (aged 22–30 years).

Oestrogen regulates lipolysis *in vitro* and *in vivo* in part through actions on the lipolytic proteins, PLIN1 and HSL 40, 41. We measured the mRNA expression of PLIN1 and HSL in whole SAT samples and while there was no difference in the expression of PLIN1, HSL expression was significantly greater in FEM SAT following E_2_ treatment relative to placebo. This suggests that decreases in cell size in the FEM region may be a result of a combination of increased recruitment of immature adipocytes (differentiation) and increased lipolysis.

Leptin was significantly increased in the E_2_ condition relative to placebo. Body composition scale weight (by DXA) was only measured at baseline, but did not change over the 2‐week treatment period. It is therefore not likely that there were significant changes in adipose tissue mass that would explain an increase in leptin. There are data in humans, animals and cells supporting oestrogen regulation of leptin. Plasma leptin concentrations vary over the menstrual cycle and the phases of pregnancy 42, 43 and oestrogen has been shown to modulate leptin production and sensitivity in rodents and humans 43, 44. In omental adipose tissue collected from non‐obese women, *ex vivo* treatment with oestradiol resulted in increased leptin secretion, which was not observed in explants from men 45. It is well accepted that oestrogen acts on total adiposity *via* interactions with its canonical receptors (ERα and ERβ) in whole adipose tissue and on mature adipocytes 46, 47, 48, 49. Further, studies in 3T3‐L1 adipocytes have demonstrated bidirectional communication between the leptin and oestrogen receptors 50. While we did not find differences in ESR1 and ESR2 expression between E_2_‐treated and placebo conditions in either AB or FEM SAT, it is possible that ERa and ERb protein were been affected. Oestrogen may also have indirect effects on leptin secretion, as leptin secretion is induced within 9 days of *in vitro* differentiation initiation in ASC 51. The increase in plasma leptin in our participants could be indirect *via* an increase in newly differentiating adipocytes in response to transdermal E_2_.

The current pilot study included a relatively small number of participants, limiting our ability to detect treatment and regional differences. Moreover, given that the physiological actions of the exogenous E_2_ treatment likely depend on time since menopause and duration of oestrogen deficiency, the fact that there was a wide range in time since menopause among our women likely added to the variability in responses. Nevertheless, these preliminary studies add novel data to the literature. We were unable to collect enough SAT in our lean participants to complete protein and activity analysis for the ERs, PLIN1 and HSL; these analyses will be required to further elucidate the effect of short‐term E_2_ treatment on these proteins.

There have been multiple studies investigating the effects of E_2_ on the adipogenic potential of various cell lines (*e.g*. bone marrow‐derived stem cells 11, 33, 3T3‐L1 fibroblasts 10 and animal tissues 9, 10). However, studies of human adipose tissue, comparing different depots and administering E_2_ *in vivo* are lacking. Our analysis of ASC isolated from the AB and FEM tissues of post‐menopausal women treated short term with transdermal E_2_ begins to fill this void and provides proof‐of‐concept for *ex vivo* analysis of clinical modulation of SAT. Future studies will include a larger number of participants and control for time since menopause.

## Conclusion

Greater femoral adiposity is associated with an advantageous metabolic profile (glucose, insulin, triglycerides and HDL‐C) 2, whereas abdominal fat accumulation is associated with insulin resistance and lipotoxicity 27, 52. After menopause women begin to store less fat in the femoral region and more fat abdominally 53, 54. It is therefore important to understand the mechanisms driving regional fat distribution. Our results demonstrate a role of E_2_ in the regulation of femoral adipogenic potential, specifically in increasing ASC differentiation. This would suggest that the loss of endogenous oestrogens at menopause may reduce the ability of FEM SAT to recruit progenitor cells to triglyceride‐accumulating cells, while AB SAT remains unchanged in this regard. Such regional changes in adipocyte lipid storage could contribute, in part, to shifts in regional adiposity to a more android distribution, resulting in the morbidities associated with menopause.

## Conflicts of interest

Authors have no competing financial interests to declare.

## Author Contribution

KCY and REV conceived of the experiments and wrote the manuscript. KCY performed cell culture work and assays. CBE processed biopsies and did cell sizing. RIP and DHB performed biopsies and RIP edited the manuscript.
